# Diagnostic accuracy of half-contrast dose bSSFP vs full-contrast dose hEPI MR perfusion imaging in patients with known or suspected coronary artery disease

**DOI:** 10.1186/1532-429X-11-S1-P8

**Published:** 2009-01-28

**Authors:** Chiara Bucciarelli-Ducci, Peter Gatehouse, Rory O'Hanlon, Jonathan Lyne, Agata Grasso, Joanna Petryka, Ricardo Wage, Winston Banya, Sanjay Prasad, David Firmin, Dudley Pennell

**Affiliations:** CMR Unit, Royal Brompton Hospital, London, UK

**Keywords:** Cardiovascular Magnetic Resonance, Perfusion Defect, Suspected Coronary Artery Disease, Diagnostic Confidence, Normal Perfusion

## Background

Non-invasive evaluation of myocardial perfusion with cardiovascular magnetic resonance (CMR) is clinically valuable in patients with known or suspected coronary artery disease CAD) but dark rim artifacts mimicking perfusion defects remain a diagnostic challenge. The perfusion protocol (pulse sequence and dose of contrast) giving highest diagnostic confidence is not yet standardised. Hybrid EPI (hEPI) is a fast sequence reducing motion artifact by its short imaging time, but with low SNR. Balanced SSFP (bSSFP) has greater SNR but previously has been reported as more prone to dark subendocardial rim artifacts: field distortion by the high-dose bolus is one suspected reason, and this distortion could potentially be reduced by using a half contrast dose, making use of bSSFP's abundant SNR. We therefore compared these 2 sequences.

## Methods

Seventeen patients were scanned at 1.5 T (Siemens, Avanto) with both protocols. All patients underwent coronary angiography (CA) and significant CAD (>50% stenosis) was detected in 6/17 patients (35%).

The dose of gadolinium contrast agent was 0.1 mmol/kg for the hEPI sequence and 0.05 mmol/kg for bSSFP, with 15 ml flush at 7 ml/s.

Centre-out hEPI (TR 5.8 ms, 30°, ETL 4, 1860 Hz/pixel) acquired 2.8 × 2.8 × 8 mm voxels over typically 360 × 270 mm FOV (adapted per patient) at TI = 110–160 ms for each of 3 fat-suppressed slices per cycle, using TSENSE (R2), typical image time 75 ms (i.e. excluding prepulses). Linear-ordered bSSFP (TR 2.6 ms, 70°, 930 Hz/pixel) acquired the same voxel size at the same TI for central of k-space for each of 3 fat-suppressed slices per cycle, using TSENSE (R2), typical image time 125 ms. The slice acquisition order was the same in hEPI and bSSFP, resulting in approximately similar image timings through the cardiac cycle in both. Slice positions in the heart were reproduced by viewing the first study while piloting the second.

The randomised scans were scored by 2 blinded experienced observers based on the 16-segment model. Perfusion scan myocardial SNR and diagnostic confidence were assessed subjectively (score from 0 = unusable to 4 = excellent). Severity of perfusion defects was graded based on its transmurality (0 = none, 1 = <25% 2 = 25–50%, 3 = 51–75%, 4 = >76%, and A = dark rim artefact). A total of 272 segments were analyzed. Sensitivity and specificity of both sequences were calculated and comparison of agreement between observers and scan methods was assessed by kappa coefficients.

## Results

The agreement between bSSFP and hEPI scans on the presence of normal perfusion, artefacts or genuine perfusion defects was 65% (k = 0.25, 95% CI: 0.17–0.33, p < 0.0001) for observer 1 and 53% (k = 0.15, 95% CI: 0.09–0.21, p < 0.0001) for observer 2.

The agreement between observer 1 and 2 on the presence of normal perfusion, artefacts or genuine perfusion defects was 83% (k = 0.51, 95% CI: 0.41–0.61, p < 0.0001) for hEPI scans and 53% (k = 0.15, 95% CI: 0.10–0.26, p < 0.0001) for the bSSFP scans.

True artefacts (compared against CA) occurred more frequently with bSSFP (59 segments, 22%) than with hEPI (14 segments, 5%) (X^2^, p < 0.001).

Using CA as gold-standard, sensitivity and specificity of bSSFP scans was 17% and 88% (observer 1), 25% and 72% (observer 2).

Conversely, sensitivity and specificity of hEPI scans was 42% and 87% (observer 1), 33% and 84% (observer 2).

Observer 1 noted a significant lower diagnostic confidence for bSSFP vs hEPI scans (p < 0.03). Both observers reported a similar diagnostic confidence for hEPI scans. The SNR of the bSSFP and hEPI were similar for both observers.

## Conclusion

Overall, bSSFP demonstrated lower diagnostic accuracy compared to hEPI. Dark-rim artifacts occurred more frequently in the bSSFP than hEPI, even when the half-contrast dose was used. Although two experienced observers were usually able to correctly identify dark-rim artefacts, using bSSFP in clinical practise in less experienced centers may not be ideal because of reduced diagnostic confidence.

